# Why Are We Scientists? Drawing Inspiration From Rita Levi-Montalcini

**DOI:** 10.3389/fncel.2021.741984

**Published:** 2022-01-21

**Authors:** Francesca Malerba

**Affiliations:** Fondazione EBRI Rita Levi-Montalcini, Rome, Italy

**Keywords:** Rita Levi-Montalcini, NGF, neurotrophin, neuroscience, women in science

## Abstract

In 2007, drawing inspiration from her previous experiments on chick embryos, Rita Levi-Montalcini, at the age of 98, proposed a new project, and a research group, in which I was included, was formed at the European Brain Research Institute (EBRI). Looking back on this experience, I can say that Professor Levi-Montalcini’s approach and the relationships she formed with my colleagues and me, contributed to my growth as a researcher. With her welcoming and warm-hearted disposition, she taught me how to consider other people’s ideas without prejudice, to reason and not to exclude any hypothesis. I also learned from her how to overcome those difficulties that are so frequent in the research field, always keeping in mind the starting point and looking toward the objective, with a factual optimism. I was just a young researcher and deeply flattered that a Nobel Laureate, with an incredible career and extraordinary life, treated me as her equal. My experience with Professor Levi-Montalcini has also provided me with a reliable path to follow, and when I encounter difficulties and challenges, I ask myself what would she have done. This approach has always helped me to move forward. Indeed, I believe the best way to celebrate Rita Levi-Montalcini as a woman in neuroscience is to recount how her exceptional example is a constant reminder as to why I have chosen to be a scientist. I hope she will always continue to be a source of inspiration for scientists in the future.

## Introduction

Rita Levi-Montalcini was born in 1909 in Turin and her life was both long and extraordinary.

When I visit schools to tell young students about Rita Levi-Montalcini, I always start by repeating the story described in the first few pages of her autobiography (Levi-Montalcini, 1988) that, in my opinion, represents a defining moment in her life. When Rita played in the park as a child, her friends used to ask her two questions: what does your father do? What is your religion? Rita had no difficulty in answering the first question: her father was an engineer, but, having been raised in a non-religious home, she was not able to respond to the second question. When she asked her father what she should say when her friends inquired about her religion, he replied: “You tell them that you are a free thinker.”

Subsequently, Rita Levi-Montalcini remained a lifelong free thinker, something of which she has given us countless examples. As a young girl, she decided to become a doctor following the premature death of her childhood nanny, against the wishes of her family. Following her expulsion from university due to the Fascist racial laws, she set up a small laboratory in her bedroom, so as not to interrupt her research. She was forced to publish through Vatican and Belgian scientific journals since Jews were not allowed to publish in other journals. Ultimately, she demonstrated that she was a free thinker upon leaving Italy (as a lone woman, in 1946) to continue her research in the United States, and when she subsequently conceived the “theory of neurotrophins,” despite it being against the current flow of ideas on nervous system development.

I have listed only a few examples here, since her biography and the story of NGF discovery are well known and described in the books “In Praise of Imperfection,” her autobiography (Levi-Montalcini, 1988) and “The Saga of Nerve Growth Factor” (Levi-montalcini, 1997). Another fascinating book, “Cantico Di Una Vita” (Levi-Montalcini, 2000), tells the story of NGF discovery by means of the letters that Rita wrote daily to her mother and twin sister (and latterly to her nephew).

In 1969, Rita Levi-Montalcini returned to Italy to manage the Centre of Neurobiology of the National Research Council (Rome), but continued to “commute” between the United States and Italy in order to monitor her laboratory at Washington University. She was awarded the 1986 Nobel Prize in Physiology or Medicine jointly with Stanley Cohen, and in 2001, she was nominated Senator-for-life by Italian President Carlo Azeglio Ciampi.

However, Rita Levi-Montalcini’s story did not simply stop following these and other prestigious awards, nor would her sense of political and social commitment be satisfied by merely monitoring experiments in the laboratory. In September 2001, at the age of 92, she attended to the “Forum Ambrosetti,” an annual meeting of industrial leaders, to present the idea of establishing a Brain Research Institute in Italy. Professor Levi-Montalcini obtained the financial support, and in 2002, together with her collaborator Pietro Calissano, she founded EBRI. The Institute became operational in 2005, through the recruitment of Italian and foreign scientists, chosen by the international scientific Committee.

**FIGURE 1:**
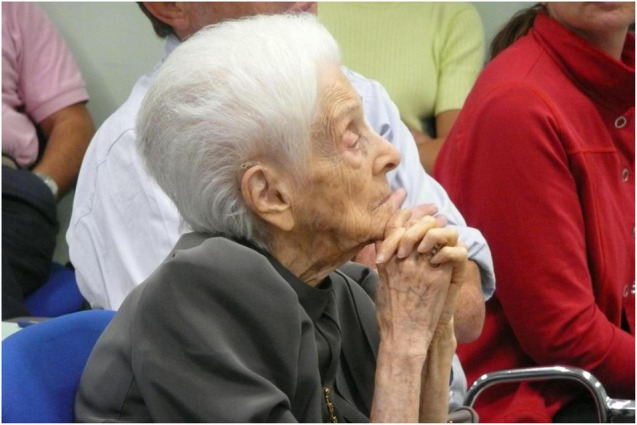
Rita Levi-Montalcini during a seminar at EBRI [Photographer Federico La Regina (EBRI) from EBRI Media Archive].

It was common to happen upon Professor Rita Levi-Montalcini, always dressed in the most elegant of outfits, when passing through the halls of the Institute located on the outskirts of Rome. Scientific life at EBRI was very lively, with meetings and seminars, often held by international scientists (Figure 1). It was in this period that I won a fellowship at EBRI.

In this article, I will describe Levi-Montalcini’s last research project, how she formulated the idea, drawing inspiration from her previous experiments on chick embryos, and how she followed the experiments. Alongside the main project, I also will describe some of her other scientific insights and ideas she conceived about “the vital role of NGF.” I will share some significant episodes during her daily working life and describe the relationship she had with her collaborators, her welcoming and warm disposition and her curiosity regarding all aspects of science and research. Moreover, I would like to describe her strong commitment to promoting the role of women in education and science, and her continuous work within science dissemination, in particular with young people and students.

Since much has been written about Rita Levi-Montalcini, and her biography and scientific career are well known to the scientific community, my contribution will be to describe the final part of her life, based on my direct experience. For this reason, I will not mention her other collaborators, who were not involved in the projects I will describe, and with whom I did not interact.

## Embryo Chick Project

In 2007, following the summer holidays, Rita Levi-Montalcini returned to EBRI with a brand new idea. Recalling her previous experiments on chick embryos, she wondered why NGF and its receptors are expressed in embryos many days before the nervous system is actually formed. She was sure that NGF must perform other functions in early chicken embryo development, besides its well-established actions on the developing sympathetic and sensory neurons, because in nature nothing is left to chance.

One might wonder how and when a 98 years old scientist, president of a fledgling research Institute, with an institutional engagement, found the time and energy to dream up a totally new project that was not in line with any research program developed in the institute.

She used to say: “Sleeping is a waste of time at my age. During the night I think” and she did not give up on “sniffing out other truffles” …

*“I think there are few things in the world as delightful as giving birth to new ideas and nurturing them*. (…) *this is one of the aspects of my work that I find captivating, a bit like a truffle dog searching for truffles, even if they are not to be found, the smell in the air is very exhilarating. I believe I have a very good sense of smell and*… *I hope to sniff out a few more truffles”* (Levi-Montalcini, 2000).

She discussed her idea with Antonino Cattaneo and considering that little was known about the actions of NGF during early embryonic stages, they decided to form a new research team. Their strategy was to block the NGF action by means of a well-validated monoclonal antibody [anti-NGF mAb αD11 (Cattaneo et al., 1988)], able to bind mature NGF with high affinity, in an earlier stage of chicken embryo development with respect to that of the nervous system, precisely at HH 11–12, according to the Hamburger-Hamilton classification (Hamburger and Hamilton, 1992). Indeed, at the embryonic stage HH 20 and HH 33–40, NGF is required for the development and maintenance of a specific population of peripheral sympathetic and sensory neurons (Hamburger and Hamilton, 1992), while NGF mRNA expression was detected initially at HH 3–5, reaching a peak at HH 33–34 (Ebendal and Persson, 1988; Baig and Khan, 1996).

The approach of “destroying” NGF effects by an anti-NGF blocking antibody recalls Rita Levi-Montalcini’s seminal immune-sympathectomy experiment: the destruction of sympathetic ganglia in mice by way of an injection of an anti-NGF antiserum (Levi-Montalcini and Booker, 1960). Immunosympathectomy, published in 1960, was recently defined as a sort of knockout *ante litteram* (Cattaneo, 2013).

In 2007, two different NGF knock-out mice were available. The “B6.129S7-Ngftm1Gne/J,” also called NGF KO, is a genetic knock-out and exhibits a short life span (about 4 weeks after birth) with delayed development and cell loss in the sympathetic ganglia (Crowley et al., 1994). Another NGF knock-out mouse, named AD11, was developed by Cattaneo’s team and was therefore available at EBRI. AD11 is a phenotypic NGF knock-out, since it was achieved by expressing the transgenic anti-NGF antibody αD11. The AD11 mouse is vital, and shows a progressive neurodegenerative phenotype resembling Alzheimer’s disease (Capsoni et al., 2000).

On the other hand, there are no knock-out chickens in existence, therefore, in chickens the only way to observe the consequences of NGF deprivation, in order to understand the related NGF function, was to block the NGF action through the use of antibodies. In Rita Levi-Montalcini’s opinion, the antibody strategy was not only the means to overcome the absence of knock-out models, but also an occasion to observe the NGF effect in different stages of embryonic development, choosing a precise time-window for the blocking of NGF. Indeed, she argued that, while the transgenic knockout gives global cumulative effects, the antibody interfering approach, that she had pioneered, allows for a much-fixed temporal regulation. A conditional NGF knock-out was indeed not available at the time.

**FIGURE 2:**
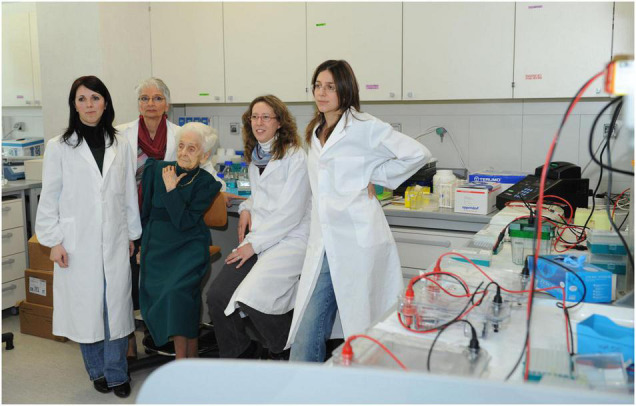
Rita Levi-Montalcini in the laboratory with her group. From left to right: Annalisa Manca, Anna Di Luzio, Francesca Paoletti and Francesca Malerba [Photographer Maurizio Riccardi (Agr Press) from EBRI Media Archive].

Once the project had been designed, Antonino Cattaneo called upon some of the researchers, already working within the context of EBRI. Annalisa Manca and Anna di Luzio were involved in embryo injections, Simona Capsoni in histology, with the technical help of Domenico Vignone, and Francesca Paoletti and myself in the carrying out of biochemical experiments. As the research progressed, the project had need of further expertise and more people were involved, but we were the initial nucleus of Rita Levi-Montalcini’s first group at EBRI (Figure 2).

I remember very clearly the first time she spoke to me. I was embarrassed and I felt completely inadequate standing there in front of her. However, those feelings disappeared after a while, when she began to question me, not about the experiments I was carrying out, nor my scientific curriculum, but about my family and where I grew up. When she heard that I came from Puglia, she told me that, as a child, she had lived in Bari due to one of her father’s job. I was immediately struck by her sweet smile and the sincere interest she showed in me.

My role in the project, together with Francesca Paoletti, was to set up and validate an ELISA to measure NGF, as a protein, in the different stages of chick embryo development. However, beyond our specific roles, my colleagues and I used to participate together in all aspects of the project as a whole. As I will describe in the coming paragraphs, we attended daily meetings with Professor Levi-Montalcini.

**FIGURE 3:**
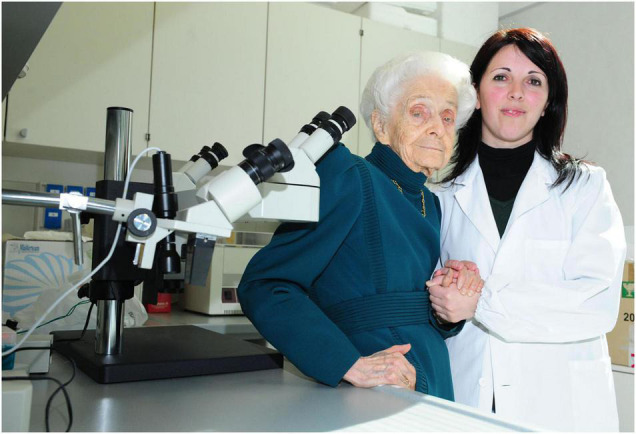
Annalisa Manca and Rita Levi-Montalcini close to the microscope with two oculars [Photographer Maurizio Riccardi (Agr Press) from EBRI Media Archive].

It was a particularly touching moment when Professor Levi-Montalcini taught Annalisa Manca how to handle/operate embryos, just as she used to do in the fifties. Despite the fact that she was almost completely blind due to a maculopathy, she came back to the laboratory to assist Annalisa, step by step, during her first injections, by using a microscope with two pairs of oculars (Figure 3).

**FIGURE 4:**
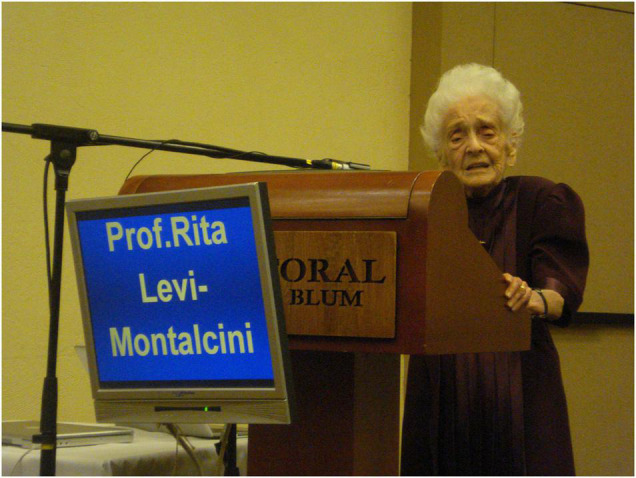
Rita Levi-Montalcini during her talk at the International NGF meeting, in Israel, 2008 [Photographer Annalisa Manca (EBRI) from EBRI Media Archive].

The preliminary results of the experiments unveiled a new NGF function: embryos treated with αD11, and not with the unrelated antibody, exhibited an inversion of the direction of the axial rotation. The final results were published in 2012 (Manca et al., 2012), and in 2008, Professor Levi-Montalcini presented our preliminary results by giving a 30-min talk and sponsoring a poster at the International NGF meeting, in Israel (Figure 4; Bradshaw et al., 2017). She set off on a long journey by air and car, to enthusiastically show the results of her work to the scientific community, as if she were a young postdoc and not a Nobel Laureate. We will never forget her zeal and passion, that drove our research in those years and long into the future.

## The Vital Role of NGF: Octopus and Fertilization Experiments

While the experiments on chick embryos were ongoing, Professor Levi-Montalcini was intrigued by the evolution of the nervous system and was studying the phylogenetic trees in relation to this.

She used to repeat that vertebrates and in particular mammals were not similar to insects, or in general to invertebrates, which develop entirely on the basis of a fixed genetic program. Conversely, mammals are able to adapt their development in a plastic manner, as she demonstrated in her “neurotrophic theory” (Levi-Montalcini, 1987), which was widely accepted and confirmed. The formation of appropriate numbers of neurons and glia is matched to the needs; plasticity also relates to other important neuronal functions, like learning and memory. Rita Levi-Montalcini always said that epigenetics is as important as genetics in mammals. Her idea of an environmental influence on vertebrate development was indeed the driving force in her discovery of NGF.

*“Only insects do not hatch until they are perfect and from that moment, neither a hair nor a cell undergoes further changes. We, vertebrates or rather primates, less perfect and less pretentious, continue to grow, some more and some less, some better and some worse”* (Levi-Montalcini, 2000).

It is important to remember that *Caenorhabditis Elegans* has a nervous system, without having NGF. On the other hand, in the nervous system of *Drosophila Melanogaster*, which has additional complexity with respect to *Caenorhabditis Elegans*, homologs of mammalian trophic factors, acting as regulators of neuronal and glial survival, were recently found (Hidalgo et al., 2011; Richardson and Shen, 2019). In particular, a structural NGF homolog, named Spatzle was found, originally discovered related to other functions in embryo development (Mizuguchi et al., 1998; Hoffmann et al., 2008).

However, it was not the fruit flies that attracted Rita Levi-Montalcini’s attention. She had known that among invertebrates, the octopus has extraordinary skills in terms of behavior and intelligence.

Indeed, octopi have evolved large and complex nervous systems and sophisticated behaviors, comparable with mammals (Hochner, 2004; Moroz, 2009). Octopi exhibit abilities such as exploring new environments, problem solving and play-like behaviors under stress-free conditions (Hanlon and Messenger, 1996; Kuba et al., 2003). Rita Levi-Montalcini was fascinated by these particular skills and asked herself whether the octopus could have an NGF-like protein, involved in its neurodevelopment. She contacted Professor Graziano Fiorito from the Stazione Zoologica Anton Dohrn of Naples, who enthusiastically agreed to a collaboration. Francesca Paoletti, Simona Capsoni and myself began experiments on different octopus’ nervous areas. Our aim was to find an NGF-like immunoreactivity through the means of simple techniques like immunohistochemistry, immunoprecipitation, ELISA and Western Blot, and by using a panel of anti-NGF antibodies. During the years that we were engaged in these experiments, the octopus genome had not yet been sequenced (Albertin et al., 2015).

Furthermore, at that time, Professor Levi-Montalcini was considering a new function for NGF, that she named “the vital role of NGF.” She imagined NGF as a sort of “organizer,” not only in relation to the nervous system, but also important in pre-embryonic life. The presence of NGF in male genital secretion was known about since the 1980s, when it had been found in the prostates of guinea pigs, rabbits, pigs and bulls, and successively, in bull, rabbit, camel, llama and alpaca seminal plasma (reviewed in Castellini et al., 2020), thus suggesting an important role for NGF in sperm function, as also mentioned by Rita Levi-Montalcini in her Nobel Lecture^1^. The expression of both NGF and its receptor in several parts of the reproductive system and its consequent involvement in sperm function led Rita Levi-Montalcini to postulate a possible involvement of NGF in oocyte fertilization by sperm. Federico La Regina and Simona Capsoni carried out a series of mice sperm and oocyte fusion experiments, both in the presence and absence of the monoclonal anti NGF blocking antibody αD11 (Cattaneo et al., 1988), the same used for the chick embryo experiments.

The preliminary experiments, both relating to octopi and fertilization projects, presented some very interesting results. Unfortunately, the experiments were not continued and the data remained unpublished, due to the fact that these projects were not funded. This was a great pity because we now know that the amazing insights gained into the “vital role of NGF” were confirmed by subsequent studies.

In 2015, the Octopus Genome was published, offering an important input to research (Albertin et al., 2015). In subsequent years, it was demonstrated that the octopus:

1) Has extraordinary sensory organs that intercept signals and integrate them in the central nervous system (Huffard, 2013; Polese et al., 2016; Stubbs and Stubbs, 2016; Di Cosmo and Polese, 2017; Di Cosmo et al., 2018).
2) Has an RNA editing mechanism, probably involved in enhancing its adaptability, in a less risky way than by changing DNA. An example of edited proteins, genetically expanded in the octopus genome, are protocadherins, important in controlling neural circuits and promoting nerve cell excitability (Albertin et al., 2012; Liscovitch-Brauer et al., 2017).
3) Exhibits adult neurogenesis, that is related to its ability to problem solve (Bertapelle et al., 2017).

However, even more surprisingly, Rita Levi-Montalcini’s intuition as regards the role of NGF in oocyte and sperm fertilization, was confirmed definitely when the article “The nerve of ovulation-inducing factor in semen” by Ratto et al. (2012) was published in PNAS in September 2012.

A protein factor, called ovulation-inducing factor (OIF), that elicits an ovulatory response in species displaying both induced and spontaneous ovulation, was known to exist in seminal plasma.

In the previously cited article, the authors purified OIF from llama e bull seminal plasma and carried out biochemical analysis to identify and study this protein. Surprisingly, Mass Spectrometry revealed a molecular mass and sequence that was identical to NGF. Moreover, X-ray diffraction data were used to solve the full sequence and structure of OIF, which confirmed the identity of both the sequence and the structure of OIF to NGF. The authors also performed crossed bioassays to test whether NGF was able to induce ovulation and whether OIF provoked neurite outgrowth in PC12 cells. Finally, they concluded that OIF in seminal plasma is indeed NGF, and that it is highly conserved across different species. An endocrine route of action of NGF elucidates a previously unknown pathway for the direct influence of the male on the hypothalamo-pituitary-gonadal axis of the inseminated female.

Undeniably, Professor Levi-Montalcini was a brilliant scientist with a great mind, despite the fact that she often declared that she was merely “reasonably intelligent” and driven mostly by intuition and imagination.

“*Imagination is more important than knowledge*”: stated Professor Levi-Montalcini quoting Albert Einstein (Viereck, 1929), and she revealed to us that this sentence had been displayed for years on her little desk in the office at the Istituto Superiore di Sanità. Einstein declared in the same interview “*I am enough of the artist to draw freely upon my imagination. Imagination is more important than knowledge. Knowledge is limited. Imagination encircles the world*,” which is the complete citation (Viereck, 1929). Likewise, Rita Levi-Montalcini often defined her approach to science, as artistic. She asserted that her artistic *modus operandi* in scientific research derived from her genetic inheritance: her twin sister was a painter, her brother an architect. She herself was a very talented illustrator. To further explore the nature of the creative process by which scientists conceive their theories, readers can refer to the bibliography (Holton, 1998; Feinstein, 2006).

Personally, I think that Rita Levi-Montalcini’s “artistic” method was the result of certain important qualities: observation and deductive logical reasoning, mixed with a holistic overview. Currently, we may take advantage of a wealth of sophisticated techniques and maybe my generation, more than those previous, tends to delegate the research answers to techniques, missing out some steps of the scientific method. Another erroneous approach of my generation is to lose the “view from above” too frequently, and to focus instead on solving a particular problem, regarding the context of the experiment. Rita Levi-Montalcini taught us to examine the question as a whole, and that each problem or negative result is there to tell us something. Sometimes we cannot understand immediately what these negative results might mean, and therefore we have to change our approach and wait for the answers to come. By way of example, we can consider the so-called “mouse effect” (Cohen et al., 1954). Professor Levi-Montalcini had found that a soluble factor isolated from sarcoma 180 and 37 caused intense proliferation of nervous fibers in chick embryos. While she was developing a bioassay in Rio de Janeiro, Levi-Montalcini found that several normal mouse tissues, used as non-tumoral control, caused a small but significant outgrowth of fibers from the ganglia (Levi-Montalcini et al., 1954).

*The mouse effect was a message I was not really capable of taking on board, since I could not help thinking that it diminished – to the extent of annulling – the significance of the induction of the fibrillar halo by S180 and S37* (Levi-Montalcini, 1988).

When she discovered the presence of NGF in mouse salivary glands, the “mystery” regarding the mouse effect was revealed, but in the meantime, Rita Levi-Montalcini had not lost heart and nor interrupted her research.

## Rita Levi-Montalcini’s Project and the Daily Team Meetings

**FIGURE 5:**
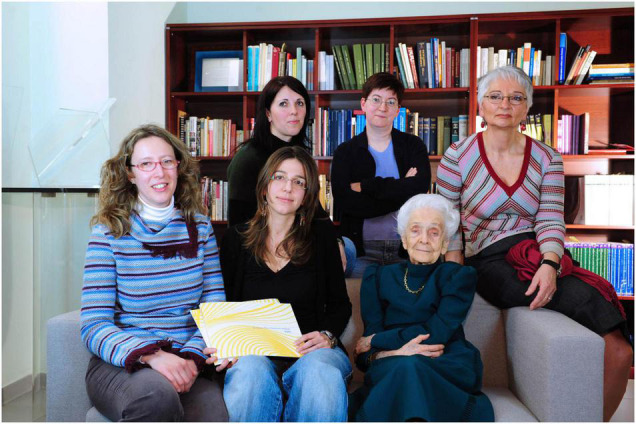
Rita Levi-Montalcini in the office with her group. Behind the sofa, from left to right: Annalisa Manca, Simona Capsoni, and Anna Di Luzio. On the sofa: Francesca Paoletti and Francesca Malerba [Photographer Maurizio Riccardi (Agr Press) from EBRI Media Archive].

Rita Levi-Montalcini used to come to EBRI almost every day and took the opportunity to have meetings with her team. We would all be seated around her on two large sofas, in her office in the original EBRI headquarters (Figure 5). First of all, she would ask to be updated on the progress of the experiments. We each took turns, by changing positions, to sit next to her because she had severe hearing loss and we had to speak close to her ear.

When we informed her of the latest results of our experiments, she used to hold our hands. I always thought that, since she had lost her sight and hearing, maybe she found a sort of compensation through that gentle contact that enabled her to create a stronger connection with us, not only mentally, but also physically. I will never forget that incredibly human gesture, that was simultaneously, meaningful, emotional, moving and the most natural thing ever.

During the first meetings that we ever had with her, she insisted that we call her “Rita” and not “Professor,” and to use the familiar form “*tu*” and not “*lei.*” (In contrast to English, in Italian there are two pronouns for “you”: the second person form “*tu*” (you) is used when speaking to someone with whom you are intimate or someone younger, and the third person form “*lei*” (he/she) when formality is required). All of us tried to adapt to this more intimate form of speech, but we could not do it! For us, she represented an eminent scientist and woman, with whom we had the fortune to know and work alongside, so it was inconceivable to address her as if she were a friend, despite our deep sense of affection. She was a little disappointed, but incredibly firmly convinced that our refusal was due to her age and not to our being awed by her remarkable personality and value! “I can comprehend” she said, “I am old and my face is full of wrinkles.”

After our daily updates on the chick embryo experiments, she was also curious to know about the other projects we were working on. During those years, Francesca Paoletti and I were working on the NGF precursor, proNGF. In particular, we were trying to obtain specific structural information about the pro-peptide, that we had found to be an intrinsically unstructured peptide (Paoletti et al., 2009). To this end, Francesca and I were trying to express proNGF in minimal medium enriched with isotopes to perform NMR spectrum acquisition. It was not a straightforward process due to the fact that proNGF is expressed in *E. coli* inclusion bodies and must be refolded. Professor Levi-Montalcini was extremely interested in our attempts to obtain the recombinant protein enriched for NMR. She was very curious about the new methods and technologies available and asked many questions about structural biology techniques. Despite the fact that she had so much to teach us, she had a constant desire to learn new techniques from us and listen carefully to our little everyday problems in the laboratory.

Science was not the only subject of discussion during meetings with Rita Levi-Montalcini. She used to ask us about our lives, our families, our dreams: Where do you live? Where are you from? Are you in a relationship? What jobs do your relatives/family do? Surprisingly, her questions were driven by a sort of scientific curiosity. My mother and my brother are a mathematician and an engineer, respectively, and my father also deals with financial mathematics. She was intrigued to understand why my entire family was involved in maths and theoretical science, whilst I had decided upon a “soft” and “wet” science, like biology. “It could depend on genetics but also epigenetic reasons, linked to your experiences” she concluded.

Professor Levi-Montalcini was also keen to know if we were satisfied with our job positions and salaries. For almost all of us, the problem was never a question of position or salary but the fact that we only had a fixed term contract, sometimes lasting mere months, since our contracts were and still are linked to the projects that have been granted. As a senator, Professor Levi-Montalcini understood the problem perfectly well and fought to obtain better conditions for Italian researchers, asking for more attention and more funding from the government for Italian research (Clementi et al., 2008).

Another occasional focus of discussion in our meetings was that of books. Professor Levi-Montalcini with the help of her collaborators read a great number of books, and when she was particularly enthusiastic about a text, she bought copies and gave them to us. Some days she gave us two or three different books, novels or essays. To our great amusement, the following day Professor Levi-Montalcini would usually ask us if we had read the books and she remained astonished when we said that we had started reading but not yet finished!

Rita Levi-Montalcini also authored books and she often liked to discuss some of the themes of her publications with us. I would like to recount one significant event in this regard. She had decided to write an essay about the “two brains”: the ancient brain, the limbic part, and the new brain, the cortex and neocortex. She said that the limbic brain, fundamental during prehistory for the safety of the Australopithecus during moments of flight from dangerous animals, has been responsible for horrible events in modern history, such as dictatorships, genocides, hate, etc. On the other hand, the neocortex would be the rational part of brain, which distinguishes humanity from beasts that are driven by instinct. She concluded that the “ancient” brain, that saved humanity in the past, would drive men to extinction in the future, if not controlled by the rationality of the “new” brain.

When she illustrated this idea, I expressed my disagreement:

“Professor, I think that instinct has some positive features: parental care, love and empathy are innate, and also important for balanced mental health. We know for example, that parental care is fundamental in avoiding that traumas are transmitted to future generations”

She looked at me with an unconvinced expression.

Some days later, Pina Moliterno, her assistant, called me on the phone:

“The Professor wants to see you”

“OK, let me call the others”

“No, she wants to see you alone”

When I went to her office and sat next to her, with my hand in hers, she said that she had being having second thoughts and had partially reversed her opinion on the limbic brain. Some instinctual properties are important.

I mentioned to Pina what she had told me, that she had changed her opinion on the basis of my words. I was 30, and a Nobel Laureate had treated me as if I were her equal. I was confused, honored and deeply moved.

**FIGURE 6:**
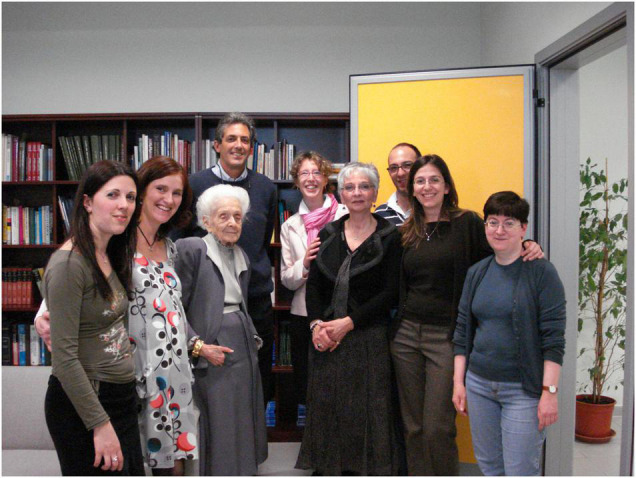
Rita Levi-Montalcini with EBRI researchers after a Christmas party. From left to right: Annalisa Manca, Raffaella Scardigli, Antonino Cattaneo, Francesca Paoletti, Anna Di Luzio, Domenico Vignone, Francesca Malerba, and Simona Capsoni (from EBRI Media Archive).

These are but a few examples of her humanity, as obviously I have a wealth of stories that deserve to be told. For example, when she gave us Christmas presents, every year she chose a different object that perfectly matched our personal style and often contained our favorite color, despite the fact that we had never declared which one it was. At the same time, we also used to give her a Christmas present, often a flowering plant, as we knew she liked them very much (Figure 6). Every year she was very happy to receive our present and always said: I will make sure that I deserve it.

In 2010, Professor Levi-Montalcini suffered a domestic accident and broke her femur. We were all extremely worried, but fortunately, the subsequent operation went well. For months afterward, Professor Levi-Montalcini was not able to move, and therefore could not come to EBRI. Naturally, this did not hold her back; instead, we went to her apartment to hold regular scientific meetings. At the time, we could sense that she was very tired, and we often wondered if our visits were perhaps too stressful for her, but her assistants encouraged us to continue to visit because the Professor wanted to see us, and did not want to interrupt her scientific activities.

## Public Life

During the years in which I was involved in Rita Levi-Montalcini’s projects, some significant public events took place and once again, she set an important example for me. She tackled everything thrown at her with elegance, assertiveness and irony.

As previously mentioned, in 2001, President Carlo Azeglio Ciampi made her a senator for life, which was a role that she did not take lightly. She never missed a session in the Senate, even though she confessed to us that she found the experience highly stressful. She said that she would prefer to be with us talking about science, but it was her duty to fulfill her institutional commitments.

Rita Levi-Montalcini was a strong advocate of increasing financial support for research. In 2006, she held the deciding vote in the Italian parliament regarding a Financial Act that was backed by the government of Romano Prodi. She threatened to withdraw her support unless the government reversed a last-minute decision to cut science funding.

“*If the Financial Act cuts the funds for research, this country is destroyed and I would not be able to vote for it.*” She declared in an interview. “*Italy has a lot of human capital and if research is not financed, the country will fall apart. We are a country that is poor in raw materials, but very rich in human capital and research is the real engine of a modern country, both in terms of social and economic repercussions.*”

Due to her support of the Prodi government, in 2007 she was the object of shameful attacks by the opposition leader Francesco Storace and his followers. Storace mockingly threatened to send her some crutches, stating that she was too old to vote and therefore represented a “crutch” to an ailing government. She was also denigrated for her Jewish origins by Storace’s supporters.

Professor Levi-Montalcini wrote a public letter, reaffirming her institutional role, her duty to participate in political decisions in the Senate, to exercise her right to vote in good conscience, in freedom and with a focus on the common needs of citizens. Moreover, she underlined that, since she had full possession of her faculties and readily continued her scientific and social activities, she did not need any crutches either physically or mentally.

When we met Professor Levi-Montalcini afterward, we expressed our indignation regarding these abhorrent insults, but with her usual calm aplomb, she said that it did not matter, that certain people did not deserve our attention and that we had more important things to attend to together.

However, Professor Levi-Montalcini took full advantage of the opportunity to get her own back on Storace through the use of irony. Shortly after, in a documentary, Watson, Nobel Laureate for DNA discovery, affirmed his previously stated view that black people are intellectually inferior to white people and that that difference is genetic. When Rita Levi-Montalcini was contacted by journalists to express her opinion, she said that “*Races do not exist. Racists do!*” and explained that the brain has the same potential in every human, while it is likely that environmental and living conditions determine the level of intelligence. She then asked:

“Are you sure that Watson expressed this horrible statement, and not Storace?”

Unfortunately, the political hatred directed at Professor Levi-Montalcini was the cause of another scandalous episode. During the 2008 public elections, the scientist went to the polling station. Since the queue was rather long, some citizens offered to let her pass in front due to her age. Not all of those waiting in line were in agreement and some declared that Professor Levi-Montalcini could wait in line along with everyone else. She was completely unfazed, saying that the people were right and that she must wait. She also refused the chair that the polling station workers had offered her.

As before, she commented to us that these matters were of little importance to her.

## Women’s Rights

Rita Levi-Montalcini’s life was hard: she was discriminated against for being Jewish and had to overcome many obstacles due to her gender. In her books (Levi-Montalcini, 1988, 2000), she describes having to fight prejudices both within her family and a work setting. As a result of her past, Rita Levi-Montalcini became a strong supporter of women’s rights, in particular she said that women and men share the same potential and skills in all jobs, but women are often overshadowed by their partners or colleagues and their names were frequently not remembered. In order to preserve the memory of some important women, she wrote the book “*Le tue antenate*” (Your ancestors), in which she illustrated the biographies of some lesser known women involved in science or social movements (Levi-Montalcini and Tripodi, 2009). The book, addressed to young students, is a great read for a general audience.

In the preface, she wrote:

*“This book is dedicated to the next generations, so that they may be aware of the fundamental scientific contributions made by their ancestors, from before the Christian era right up until the twentieth century, a significant period, in which being of the female gender was considered an obstacle to any type of intellectual development. Women were long excluded from important areas of society, on some occasions, the wisest were even accused of witchcraft and burnt at the stake. In many cases, the female contribution has never been fully recognized, attributed to the influence of fathers, brothers or husbands: of figures, always belonging to the male gender. In reality, throughout the ages and up until the present day, women have contributed to scientific development in equal measure to men, while also playing the role of wife and mother.*”

Rita Levi-Montalcini also established a foundation to assist African girls in studying scientific subjects, in particular medicine or nursing sciences by granting fellowships in European Universities.

Considering that Rita Levi-Montalcini was very active in scientific dissemination, paying particular attention to the younger generations, at EBRI we are committed to continuing her work, by organizing informative scientific meetings with general public and outreach lessons in schools.

## Discussion

In the previous paragraphs, I have described Rita Levi-Montalcini’s most recent research projects, I have detailed a few of the most relevant episodes of my daily life with her (there are many other stories that remain to be told), described how she used to interact with her collaborators, her commitment to public and political life, and the way in which she faced up to problems. I would like to remind everybody that I met her when she was 98 years old.

What did I learn from her?

1) Enthusiasm and competence in research

*“In order to do your job in the best possible way, you need enthusiasm and competence. Only having one of these is insufficient.*”

Rita Levi-Montalcini also used to cite a Primo Levi quotation from his novel “The Monkey’s Wrench” that summarized what her job meant for her.

*“If we exclude prodigious and individual moments that fate presents us with, loving our work (which unfortunately is the privilege of few) is the best concrete definition of happiness on earth”* (Levi, 2017).

2) Curiosity and open doors

Rita Levi-Montalcini was curious about each and every aspect of life and about the people she interacted with, without any distinction of cultural education and social background, because every person was a potential source of inspiration and personal growth. She always left the door open for everyone. After all, is not being able to listen, observe and show curiosity without prejudice and dogmas, while employing honesty and integrity, part of the basic principles of the scientific method?

3) Learning from negative results

“*Do not fear difficult times, the best results come from there.*” Misleading or negative results are not a waste of time, but the answers to questions that we will eventually comprehend, if not immediately, then later on. In any case, negative results can point us in the right direction, without being discouraged. She taught us to maintain “the vision from above,” keeping of the objective in mind, without forgetting the starting point.

4) Intelligence and generosity.

I greatly admired Rita Levi-Montalcini’s deep and dynamic intuition and holistic approach to all scientific questions. It was a rare gift that made her a very special person. During our meetings, we were often entranced by her observations and her creative approach that was always centered on the issue in question. On the other hand, Rita Levi-Montalcini took genuine care of us, and was interested in our experiments, opinions and projects in science and in life in general. Often the Professor showed astonishment in the face of our admiration and gratitude. She frequently declared that she was not special, but merely propelled by willpower, hard work and a little bit of good fortune. Sometimes I ask myself if Professor Levi-Montalcini did not fully realize her enormous worth as woman and scientist or, simply, that the people who possess great value somehow ignore this great value themselves. This was yet another lesson imparted by Rita Levi-Montalcini: be wary of arrogant people, since great people always display humility, generosity, attentiveness and availability.

5) Optimism and looking forward

**FIGURE 7:**
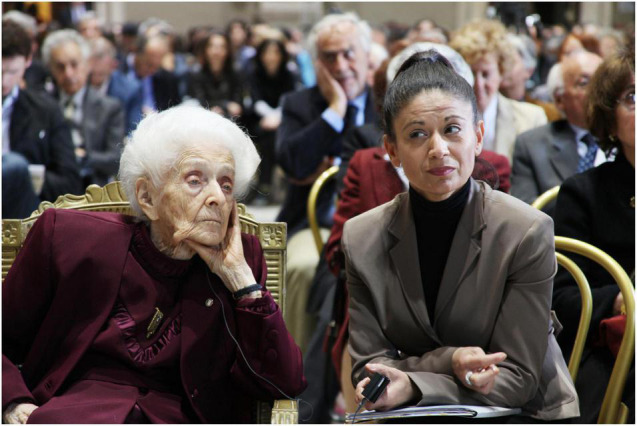
Rita Levi-Montalcini with her assistant Pina Moliterno, during the workshop “The Brain in Health and Disease,” held in the Campidoglio, Rome, on 22nd April 2019, in celebration of her 100th birthday (EBRI Media Archive).

In a video, shot in Israel, during the previously mentioned NGF meeting, in answer to the question “Have you any regrets?” she answered “never!” immediately and decisively. When, during an interview at EBRI in 2007, Moses Chao asked her what was the happiest time in her life, she replied “this one!” (Chao, 2010). In 2009, just before her 100th birthday, while being questioned by Paolo Giordano, an Italian writer and physicist, Rita Levi-Montalcini said that, despite her age, she was continuing to look toward the future with confidence (Figure 7).

If I re-read all the points that I have listed, I think that Rita Levi-Montalcini’s teachings are not only useful for scientists, but can offer an example to anyone who seeks satisfaction in life by doing something good for themselves and the next person. Being a scientist implies not expressing opinions before having studied the problem deeply, observing, postulating a hypothesis and verifying it through the appropriate checks. We should avoid prejudices and dogmas, instead relying on deduction and induction. Following this reasoning in our everyday lives can help us make important choices, especially in darker moments.

In an interview, a journalist asked me if I had learnt more from Rita Levi-Montalcini the scientist or the woman. My answer was that I could not divide Professor Levi-Montalcini the scientist from the woman.

Rita Levi-Montalcini was a scientist with the grace, delicacy, sensitivity and elegance of a woman. What is more, she was a woman embodying the principles of the scientific method in all aspects of her life. She possessed patience and tenacity, as a consequence of her life experiences, because, as she loved to repeat “*those who are full, gain less satisfaction from food than those who are hungry, and women are hungry because men have kept us out of many aspects of social, political, scientific life for centuries.*”

When I was writing this paper, I undertook some interviews with my colleagues. The question “Why did you decide to become a scientist?” seemingly simple and obvious, was met with some hesitation. For the most part, they needed time and some explanation before attempting to remember why and answer. Their responses were quite varied and interesting, but the intended goal for this “experiment” was to investigate the feelings that accompanied the answers.

*I can say that the only ideal I worked for was that of helping others and perhaps this is why research has given me much more than I could have hoped for* (From an interview).

*Considering in retrospect my long journey, and that of my peers and colleagues and the young recruits who have joined us, I can affirm that in scientific research, neither the degree of intelligence nor the ability to execute and carry out the task at hand perfectly, are essential factors for success and personal satisfaction. In both cases, total dedication and closing our eyes to difficulties are actually more important: in this way, we can face problems that others, who are more critical and more precise, would not face* (Levi-Montalcini, 1988).

*I wish young people the same luck that led me to lose interest in my own person, but to always pay great attention to everything around me, to everything in the world of science, without neglecting the values of society* (From an interview).

*Every cell, nerve cell, particularly in the brain, is such a marvelous object to study. (.) So I had all the reasons to want to work in this way, not as a scientist, but in order to see beauty* (Chao, 2010).

These are Rita Levi-Montalcini’s answers to this question.

In my opinion, scientific research is not simply a job like any other. Being a scientist is also a responsibility, not only with respect to the data we produce and share with the community, but also to those people waiting for answers from science, in particular patients, if we are referring to biomedical research. And research is not a job like any other, because you learn to collect failures and frustrations, and yet to keep going ahead.

In hard times, when we have no funds, conflicting results, many failed attempts and we are feeling frustrated and over focused on particular problems, therefore losing the “vision from above,” maybe we should close our eyes and call to mind the reason why we decided to become scientists. While considering the answers of my colleagues I have realized that we often forget this point. During difficult moments, maybe it could be useful to think of Rita Levi-Montalcini’s example and then look forward to the future with confidence, as she always did during her life.

I hope Rita Levi-Montalcini will continue to be a source of inspiration for scientists and beyond, and that my testimony in this article can play a small part in this.

*“Life does not end with death. What you pass on to others remains. Immortality is not the body, which will one day die. I don’t care about dying. That does not matter*… *of importance is the message you leave to others. That is immortality.”*

## Author Contributions

FM designed and wrote the manuscript.

## Conflict of Interest

The author declares that the research was conducted in the absence of any commercial or financial relationships that could be construed as a potential conflict of interest.

## Publisher’s Note

All claims expressed in this article are solely those of the authors and do not necessarily represent those of their affiliated organizations, or those of the publisher, the editors and the reviewers. Any product that may be evaluated in this article, or claim that may be made by its manufacturer, is not guaranteed or endorsed by the publisher.

## Footnotes

1
https://www.nobelprize.org/prizes/medicine/1986/levi-montalcini/lecture
